# Palliative care education and knowledge transfer into practice – a multicenter survey among medical students and resident physicians in Germany using a mixed-methods design

**DOI:** 10.3205/zma001682

**Published:** 2024-06-17

**Authors:** Marie-Christin Dronia, Kim Dillen, Frank Elsner, Manuela Schallenburger, Martin Neukirchen, Anna Hagemeier, Stefanie Hamacher, Axel Doll, Raymond Voltz, Heidrun Golla

**Affiliations:** 1 University of Cologne, Faculty of Medicine, Cologne, Germany; 2 University Hospital, Centre for Palliative Medicine, Cologne, Germany; 3 RWTH Aachen University, Medical Faculty, Clinic for Palliative Medicine, Aachen, Germany; 4 Heinrich-Heine University Düsseldorf, Medical Faculty, Interdisciplinary Centre for Palliative Medicine, Düsseldorf, Germany; 5 University Hospital Düsseldorf, Centre for Integrated Oncology Aachen Bonn Cologne Düsseldorf (CIO ABCD), Düsseldorf, Germany; 6 Heinrich-Heine University Düsseldorf, Medical Faculty and University Hospital Düsseldorf, Department of Anaesthesiology, Düsseldorf, Germany; 7 University Hospital Cologne, Institute for Medical Statistics and Bioinformatics, Cologne, Germany; 8 University Hospital, Centre for Integrated Oncology Aachen Bonn Cologne Düsseldorf (CIO ABCD), Cologne, Germany; 9 University Hospital, Centre for Health Services Research Cologne (ZVFK), Cologne, Germany

**Keywords:** palliative medicine, curriculum, Germany, medical edcuation, medical residency

## Abstract

**Objective:**

In 2009, Palliative care was incorporated into the medical curriculum as Cross-Sectional Subject 13 (QB13) by means of the revision of the Medical Licensing Regulations for Physicians. The aim of this study was to determine the strengths and deficits of QB13 student education for palliative care in clinical practice in a multi-centre setting and to identify potential for improvement.

**Methods:**

Online questionnaires filled out by medical students during their Practical Year (PY) and resident physicians from the university hospitals in Aachen, Düsseldorf, and Cologne were descriptively analyzed using SPSS; free-text responses were categorized and quantified. Semi-structured interviews with the resident physicians (using a mixed-methods design) were analyzed through content analysis. Emerging categories were quantified.

**Results:**

Analysis of 130 fully completed questionnaires and 23 interviews revealed that participants particularly benefited from patient- and practice-oriented small-group sessions for their clinical work. Despite some university-specific differences, the PY students identified a need for training in end-of-life-care, while resident physicians saw a need for training primarily in dealing with patients and their relatives. They also reported deficits in transferability.

**Conclusion:**

QB13 should be organised in cross-university curricula and provide sufficient resources for practical-oriented small-group teaching. Based on the "unit of care", besides caring for palliative patients, dealing with patients’ families should also be an education focus. To improve transferability into clinical practice, students should be actively involved in the care of palliative patients.

## Zusammenfassung

**Zielsetzung:**

Im Jahr 2009 wurde Palliativmedizin nach Vorgabe der ärztlichen Approbationsordnung als Querschnittsbereich 13 (QB13) in das Curriculum Humanmedizin aufgenommen. Ziel dieser Studie war es, Stärken und Defizite der studentischen Lehre des QB13 für die palliativmedizinische Versorgung im klinischen Alltag multizentrisch zu ermitteln und Verbesserungspotenzial abzuleiten.

**Methodik:**

Die von Studierenden im Praktischen Jahr (PJ) und Ärzt*innen in Weiterbildung (ÄiW) der Unikliniken Aachen, Düsseldorf, Köln ausgefüllten online-Fragebögen wurden mittels SPSS deskriptiv statistisch ausgewertet; Freitextantworten wurden kategorisiert und quantifiziert Die leitfadengestützten Interviews mit den ÄiW (Mixed-Method-Design) wurden inhaltsanalytisch ausgewertet. Entstehende Kategorien wurden quantifiziert.

**Ergebnisse:**

Die Auswertung der 130 vollständig ausgefüllten Fragebögen und der 23 Interviews ergab, dass die Teilnehmenden für ihre klinische Tätigkeit insbesondere von patient*innen- und praxisnahen Kleingruppenveranstaltungen profitierten. Bei z.T. universitätsspezifischen Unterschieden wurde ein Hauptschulungsbedarf von den PJ-Studierenden für die Terminalphase, von den ÄiW vor allem im Umgang mit An- und Zugehörigen gesehen. Letztere beklagten zudem Defizite in der Transferleistung.

**Schlussfolgerung:**

Der QB13 sollte in universitätsübergreifenden Curricula organisiert werden und hierbei genügend Ressourcen für praxisnahen Kleingruppenunterricht vorhalten. Angelehnt an die „unit of care“ sollte neben der Versorgung von Palliativpatient*innen auch der Umgang mit An- und Zugehörigen einen Lehrschwerpunkt darstellen. Zur Verbesserung der Transferleistung in den klinischen Alltag sollten Studierende aktiv in die Versorgung von Palliativpatient*innen einbezogen werden.

## Introduction

In light of described deficiencies in medical student education and social need [1], [2], [3], palliative medicine was included in the German medical curriculum as Cross-Sectional Area 13 (QB13) by means of the revision of the Medical Licensing Regulations for Physicians (ÄAppO) [4]. The subject catalogue of the German Society for Palliative Medicine (DGP) served as an orientation guide. Six teaching contents were recommended (basics, treatment of pain and other distressing symptoms, psychosocial aspects, ethical/legal issues, communication and teamwork/self-reflection) with a target scope of 40 teaching units (TU) of 45 minutes each [5]. The design and didactic implementation are at the discretion of the respective faculty and are heterogeneous [6], [7], [8], which is also reflected in differences in palliative medicine education at the participating medical faculties.

Despite generally positive teaching evaluations [9], [10], [11], [12], there are challenges, such as the discrepancy between the high number of students and the question of feasible patient contact, as well as between the number of people who require palliative care in their lives and the disproportionately low proportion of teaching in the overall curriculum [13], [14]. The use of simulated patients [15] and existing capacity for palliative medicine during the practical year (PY) [16] are not sufficient to counteract deficiencies in the application of palliative care knowledge [17], [18]. 

Internationally, palliative care is increasingly recognized as important for medical education [19], [20], which is partly reflected in the development of very good palliative care courses in terms of content and structure [21]. However, unlike in Germany, palliative medicine is not yet a mandatory part of the curriculum in many countries, leading to even more heterogeneous structures in education in terms of scope and implementation at individual faculties [22]. In addition, international surveys reveal deficits in knowledge about what palliative care can provide, particularly in the area of spiritual care [20], dealing with end-of-life situations [23], [24] and dying [25].

The present study addresses the following questions:

- How do medical students during their practical year (PY) and resident physicians evaluate palliative care education at different locations?
- Do they feel prepared for palliative care in their clinical practice through education?
- To what extent is the content taught successfully transferred into practice?
- How do they experience the current palliative care structures?

## Material and methods

### Study design

This is a multi-centre cohort study (medical faculties of the University of Cologne, Heinrich Heine University (HHU) Düsseldorf, Rheinisch Westfälische Technische Hochschule (RWTH) Aachen) with the collection of quantitative (PY students, resident physicians) and qualitative (resident physicians) data (mixed-method design). The questionnaire covered aspects of all research questions; the question regarding the transfer of knowledge into practice with certainty in the care of palliative patients were addressed and deepened in the in-depth interviews with physicians already in the medical profession.

### Setting and sample

The design of QB13 has specific features for the participating university hospitals in Aachen, Düsseldorf and Cologne: In Aachen, QB 13 takes place as “Palliative Medicine System Block” in the 7^th^ semester and is structured into lectures (12 TU) and a small-group practical course (8 TU). Cumulatively, mandatory education in palliative medicine, combined with components in the “Pain System Block”, amounts to 28-30 TU. In addition, palliative care competences can be acquired in elective courses (“The patient as teacher”, “pain therapy, grief, ethics and communication”) [26]. In Düsseldorf, QB13 was implemented as part of a fundamental restructuring of the medical curriculum [3]. Since then, the compulsory education has been further developed and now includes lectures and seminars (15 TU), an e-learning programme (4 modules, approx. 6 TU) and bedside teaching [27]. The voluntary elective courses in Düsseldorf focus on “communication in borderline situations”, “teamwork in palliative care”, and “dealing with requests for death” (28 TU/semester) [https://www.uniklinik-duesseldorf.de/patienten-besucher/klinikeninstitutezentren/palliativmedizin-izp/lehre]. The “open source” teaching materials available in Düsseldorf were not used by the other participating universities in this study. In Cologne, 40 TU are provided as a mandatory palliative medicine curriculum in QB13, distributed as follows: Lectures (21 TU), seminars on changing treatment goals, ethical decisions, advance medical directives, dying phase (9 TU) and communication training in small groups with simulated patients (10 TU) [28].

People who were students in their practical year or resident physicians in the first three years of their professional careers and and had completed QB13 at one of the participating universities were eligible to take part in the survey. The time limit of the first three professional years was intended to ensure that the resident physicians were already independently practicing medicine, but not too much time had passed since their student education in QB 13.

PY students (approx. 425 PY students/cohort, PY students in Aachen, Düsseldorf and Cologne combined) were exclusively surveyed using an online questionnaire. Recruitment took place through the e-mail distribution list of the respective deanery. Structured in-depth interviews were conducted with the resident physicians until the content was saturated (in relation to subsequent recruitment) to give particular weight to this dual perspective (not only evaluating courses but also the transfer of knowledge). To prepare for the interview, the residents physicians completed the online questionnaire to be able to elaborate on specific aspects during the interview. They were recruited through the university hospitals’ e-mail addresses and by being approached verbally by the employees involved in the study.

### Data collection instruments and implementation 

The questionnaire was designed based on a literature research [19], [29], [30] and further developed in discussion rounds with the study management and representatives of the participating palliative care centres. The questionnaire comprises five thematic complexes with 22 (PY students) and 25 (resident physicians) questions (19 questions identical, 3/6 questions group-specific) (see attachment 1 and attachment 2 ). After generating the questionnaires on the online platform lime-survey (version 3.28.56+230404), a pre-test was conducted by employees of the Centre for Palliative Medicine at Cologne University Hospital. The feedback was discussed with the study management (HG) and implemented accordingly, followed by anonymous completion of the questionnaires between 06/21 and 07/22. Two reminders were sent after six and 12 months.

The in-depth interviews were conducted using a guide based on “the global IMEP (International Assessment of Medical Education in Palliative Care) initiative”. This guide, already used internationally to evaluate education in palliative care, was translated into German [31] and comprises 12 questions (see attachment 3 ). The interviews took place between 07/21 and 07/22, were conducted web-based and recorded using integrated audio software. After verbatim transcription [32] the anonymised transcripts were imported into MAXQDA Analytics Pro 2022.3.

The project was assessed favourably by the ethics committees of the three participating medical faculties (Aachen: EK 222/21, Düsseldorf 2021-1530, Cologne 21-1094). A written informed consent was obtained from all study participants, which contained a corresponding declaration regarding applicable legal data protection regulations.

### Data analysis

The data from the online questionnaire survey were imported into the statistical software SPSS (version 28.0.1.1 [10]) by IBM and analysed descriptively and statistically. Incomplete questionnaires were taken into account in the analysis by declaring the unanswered questions as missing. For the analysis of free-text responses, a category-oriented analysis of statements was conducted using inductive categorisation. Initially, broad groupings were formed, which were then divided into overarching meaningful categories based on a descriptive analysis of frequencies [32] (see attachment 4 ).

The content analysis of the in-depth interviews was conducted using MAXQDA Analytics Pro 2022.3 according to recommendations for focussed interview analysis by Kuckartz [32] based on inductive category formation. The category system was first differentiated and condensed using the interview guide (see attachment 5 ). This was done through a consensus process by MD and KD, focusing on differences and difficult, ambiguous passages to achieve agreement on appropriate coding. Based on this the category system was further developed and finalised (see attachment 5 ). MD then coded the entire data. The results were presented to KD and HG.

The Code Matrix Browser and the Code Relation Browser in MAXQDA were used to quantify the qualitative data collected in the interviews. These methods were used to analyse both the code frequencies in the data and their co-occurrence.

## Results

### Sample

A total of 161 questionnaires were returned (response rate of 19%), of which 130 (81%) were fully completed (PY students 82%; N=106, resident physicians 18%; N=24). 23 resident physicians also participated in the interviews (average duration 28 min (19:23-45:04 min). Characteristics of all participants can be found in table 1 (Tab. 1) and table 2 (Tab. 2).

The quotes mentioned in the following paragraphs are exclusively from the in-depth interviews (listed in detail in attachment 6 ).

### How is palliative care education evaluated at the participating medical faculties?

When evaluating the proportion of palliative care courses in the overall curriculum after the introduction of QB13, the questionnaires showed a roughly balanced picture (N=143): 44.1% (N=63) rated the proportion as exactly right, while 50.3% (N=72) considered it to be too low.

**Figure 1:**
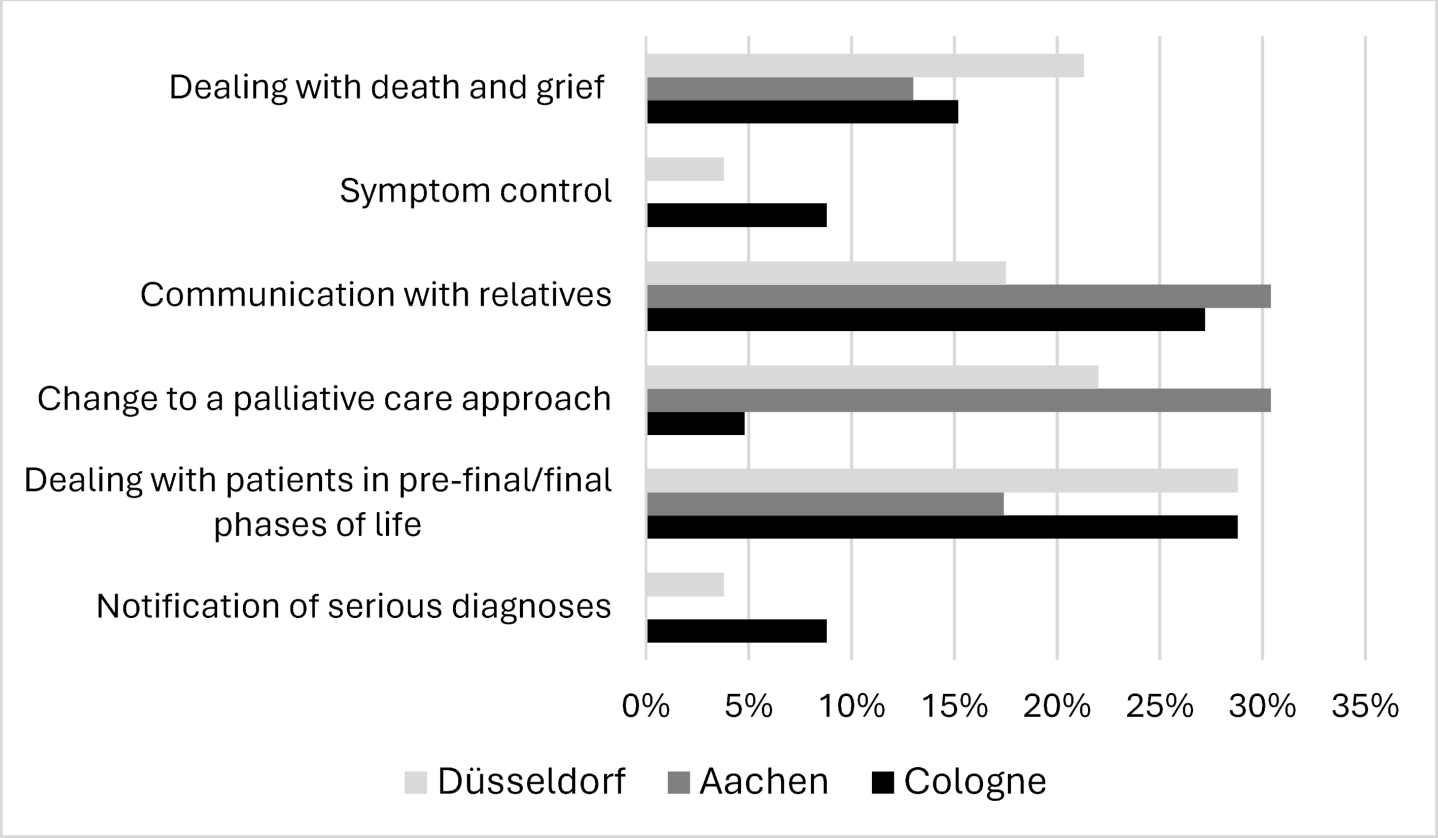
Information provided by medical students during their practical year (PY) regarding inadequately covered or missing content in palliative care education across universities

**Figure 2:**
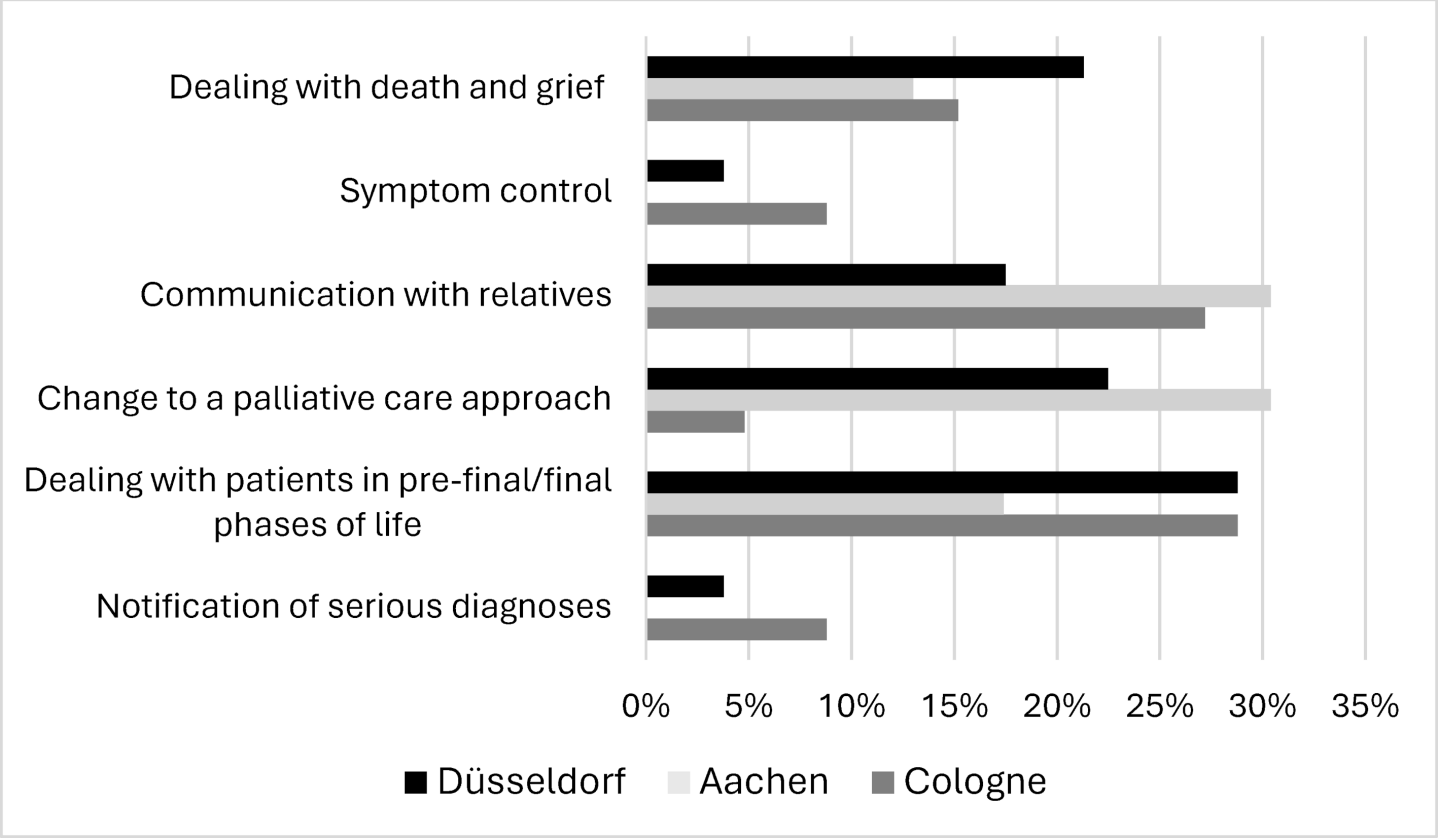
Information provided by resident physicians regarding inadequately covered or missing content in palliative care education across universities

71.3% of participants stated in the questionnaires that they missed content in palliative care education. Among the PY students, the most commonly mentioned topics were dealing with patients in the dying phase, conversations with relatives, coping with death and grief and changing a treatment approach (see figure 1 (Fig. 1)). In contrast to the PY students, the resident physicians additionally assessed symptom control as lacking or insufficiently treated (see figure 2 (Fig. 2)). University-specific differences were evident: while the topic of changing a treatment approach was rarely considered insufficiently by the PY students in Cologne (4.8%), this was much more frequently the case in Aachen (30.4%) and Düsseldorf (22.5%).

### Do PY students and resident physicians feel prepared for palliative care in clinical practise?

According to the questionnaire survey, practical events with a small number of participants (small group lessons, seminars, communication lessons, e.g. using drama patients) were attributed the greatest importance by PY students and residents physicians. This was confirmed in the in-depth interviews; these formats, in particular, help to provide adequate care for patients with incurable advanced diseases and dying patients in clinical practice (quote 5-6). Lectures and virtual formats were rated as less helpful. A quarter of the interviewed residents physicians (N=6) and 3.8% of PY students (n=5) had very valuable practical experiences in palliative care during an internship or a PY term.

### Is the transfer of the content into practice successful?

When evaluating the transfer performance, almost all participating resident physicians (N=17) emphasised that they had learned basic palliative care skills through the medical curriculum – especially through the courses with practical relevance (quote 7-9) and that they had reduced fears and inhibitions regarding dealing with patients with incurable diseases or dying patients (quote 10-12). Two-thirds of the physicians stated that they were usually able to give palliative patients justice in their everyday inpatient work (N=16). However, deficits were noted in the concrete implementation of the learned content in clinical practise (quote 13-14). This mainly included three aspects:

### Symptom control and changing therapy approach

While symptoms such as pain, nausea/vomiting and dyspnoea were generally considered to be adequately taught (quotes 15-17), this was not the case for symptoms such as malnutrition, constipation, delirium, and the use of psychopharmaceuticals in a palliative care setting (quotes 18-20). The implementation of a change in therapy approach was also seen as a hurdle in clinical practice (quote 21-22).

### Communication

The majority of participants stated that they felt confident talking to patients about approaching death (75.4% rated their ability as 4 or 5 on the Likert scale; (quote 23-24)). The interviewed resident physicians (N=23) also expressed this for the prognosis disclosure (quote 25). However, difficulties would particularly arise in communication with relatives (Quote 26). Most resident physicians used the word “death” consciously (N=16), while about a third stated using paraphrases (N=9) or avoided it completely (N=6) (quote 27-29). Training in communicative skills was perceived as essential preparation for entering the profession (quote 30). Professional activity and learning from role models (colleagues, superiors) had contributed to even more confidence over time (quote 31-32). However, it is not possible for all resident physicians to gain independent experience, as some supervisors primarily took over these conversations themselves (quote 33-34). The deficit was even more serious among PY students: Only 1.7% and 9.2% stated being involved in the treatment of patients with a palliative care concept or respectively in the delivery of serious diagnoses.

### Dealing with dying

When dealing with patients in end-of-life situations, resident physicians felt uncertainty of a practical (appropriate symptom management (quote 35)) and emotional nature. In their opinion, this was due to dying and death being insufficiently addressed in medical education (quote 36-37). They gained initial experiences during their clinical work (quote 38-39). Many resident physicians felt emotionally and psychologically burdened when their patients died (quote 40). Only a few were aware of in-clinic support services (quote 41-42), the affected doctors usually turned to colleagues or their private environment (quote 43-44). The interviews stressed that they wish for more professional support in such situations (quote 45). At the same time, they had the impression that some of their superiors did not even want them to engage in dialogue and self-reflection (quote 46).

### Suggestions for improvement in education and further training

From the perspective of the resident physicians in the first three years of their careers, palliative care education should have more clinical relevance and more practical teaching units (quote 50). They wished for palliative care, including accompanying patients until death, to be an integral part of medical education (quote 51). In addition, participants stressed the need for independent chairs for palliative medicine at all medical faculties, specialized training in palliative medicine and the expansion of palliative care guidelines (quote 52-53).

### How do PY students and resident physicians experience the current palliative care structures?

**Figure 3:**
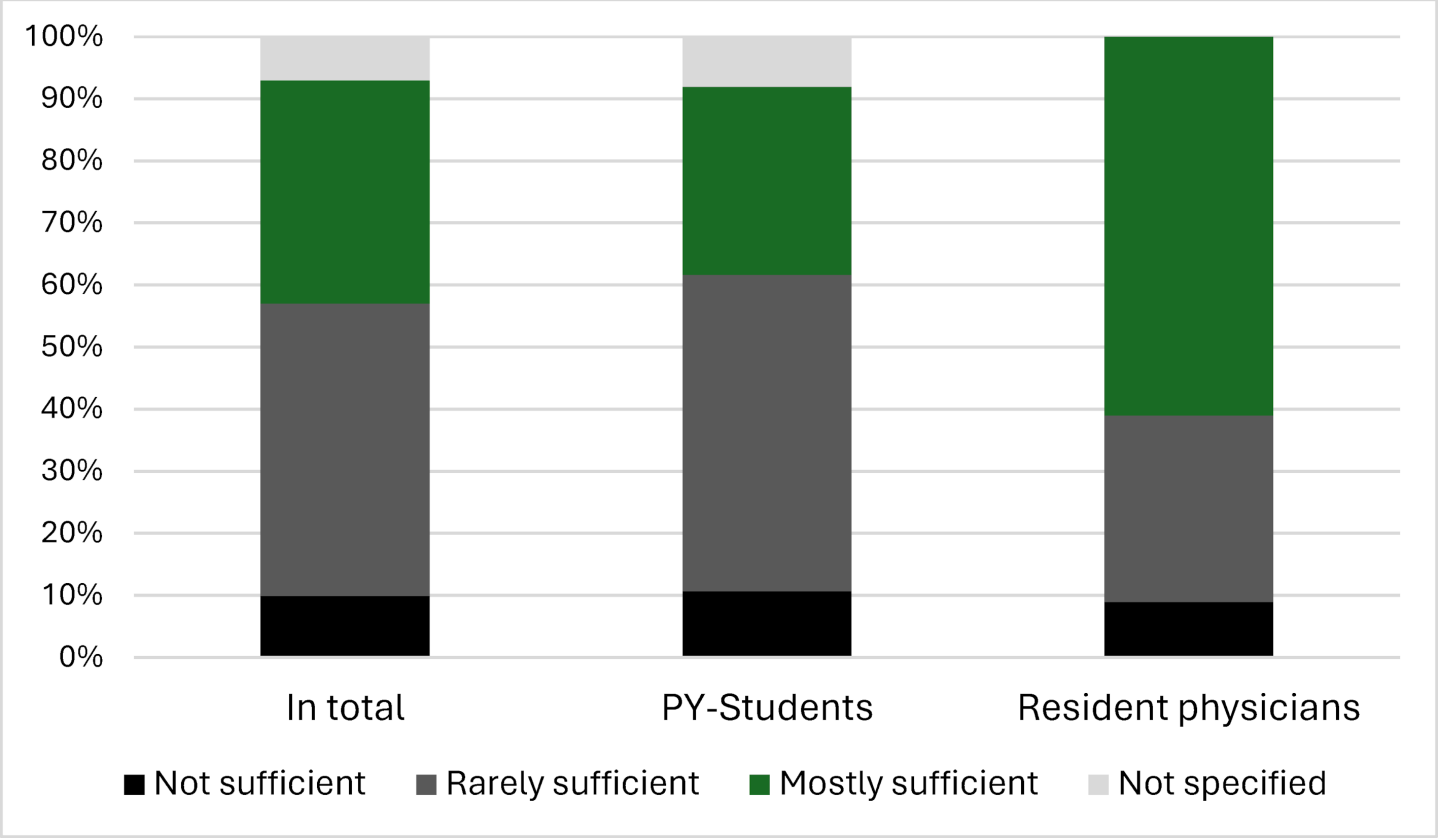
Assessment of the care provided to patients with palliative care needs in an inpatient setting by PY students and resident physicians

50.6% of the PY students and 96.2% of the resident physicians had already had contact with palliative care structures (palliative care unit, palliative care service (PMD), hospices, specialised outpatient palliative care (SAPV)). In-depth interviews particularly stressed positive experiences with SAPV and the PMD (quotes 1-3). Providing care for patients in need of palliative care was frequently encountered in the daily routine of the resident physicians (N=17). However, according to the questionnaire, the majority of both PY students and physicians rated the palliative care for the patient in an inpatient setting as unsatisfactory (see figure 3 (Fig. 3)). The participants most frequently associated the need for a palliative treatment concept with the treatment of incurable diseases using a non-curative treatment approach (23.1%). The type of disease played a minor role here (9.9%), with symptom burden (36.2%), disease stage (26.3%) and the patient's wishes (20.8%) being a more significant factor. The interviewees often felt unsure about psychological or spiritual counselling and tended to refer these aspects to specialized professionals (quote 4).

Participants talked about the following visions for the future development of palliative care in Germany: The establishment of palliative care services in all hospitals and the expansion of specialised outpatient palliative care services were universally considered necessary by the participants (100% each); the desire for independent palliative care units was also strongly stressed (94.5%). An unbureaucratic and timely transition to the necessary outpatient palliative care structures, in order to be able to meet patients’ wishes (quote 54), was considered particularly important, as was better and earlier access to palliative care, especially for non-oncological patients (quote 55). Health policy aspects such as a larger lobby for greater advocacy efforts to secure higher budgets and foster interdisciplinary collaboration (quote 56-58) were also mentioned. They also emphasized the importance of social discourse on end-of-life matters and increased awareness (quote 59). 

## Discussion

This study evaluated palliative care education at three German universities from the perspective of PY students and resident physicians. It focused on the transfer of knowledge into clinical practice and assessed the adequacy of existing palliative care structures.

### Evaluation of teaching

After the introduction of QB13, students develop a basic understanding of palliative care principles (citation 47-49), [11]. However, there remains a lack of confidence in the daily implementation of palliative care aspects [17]. This perceived deficit was confirmed among PY students and resident physicians in their first three years in this study. This deficit may explain why around half of the participants consider palliative care education to be underrepresented in the overall curriculum (50.3%), despite the recommended amount of time is essentially implemented. While expanding palliative care content within the comprehensive medical curriculum is challenging [7], a focus on practical teaching in small groups [33], [34], case-based, multiprofessional [11], and bedside teaching [35] appears essential to facilitate better knowledge transfer [36], [37]. Communication training during and beyond medical studies is crucial, as emphasized nationally and internationally [19]. Furthermore, to enhance knowledge transfer, students should be actively involved in the care of palliative patients, including those at the end of life [38], a practice that seems lacking according to this and other surveys [17], [18]. This would certainly also improve the concrete clinical implementation of symptom-controlling treatment, for which a good theoretical basis is provided in courses, but has not been effectively pursued in practice. Lectures scored significantly lower in this survey in terms of their influence on the ability to provide care compared to practical teaching units/small group courses (e.g. University of Cologne: Lectures 14.9%, Competence Course change of therapy goal 30.4%, communication training 21.4%). Nevertheless, they remain the leading teaching method [39] and should be deferred in favor of other formats. E-learning formats have also been trialled in palliative care [40], and were particularly useful during the SARS-CoV-2 pandemic [41]. However, our study results indicate these formats were not considered essential by learners for transferring knowledge into practice. The differently perceived deficits in the palliative care curricula of the individual universities speak in favour of the need for more intensive exchange between faculties, such as regular meetings to derive common learning objectives, shared use of teaching materials and proven small group formats, continuous curriculum evaluation and analysis, and university-wide mandatory curricula. This has already been implemented in Australia and the UK [42], setting a precedent as international palliative medicine teaching structures are considered highly heterogeneous [38]. In addition to student teaching, it will also be necessary to evaluate in-service palliative care training and further education programmes.

### Importance of palliative care

The increasing importance of palliative care nationally and internationally [19], [43], [44], [45] is also reflected in the present study: Students increasingly learn about palliative care approach and content during their education. The treatment of patients with a palliative care treatment approach and cooperation with general and specialised palliative care structures [46], [47] plays an important role in the everyday clinical practice of most resident physicians. The existing disparity between the increasing need for adequate palliative care and the existing structural possibilities [4], [16] is consistent with the statements made by the participants in this survey. Despite the expansion of inpatient and outpatient palliative care structures in Germany in recent years [48], almost all respondents, in line with previous study findings [49], [50], [51], [52], [53], advocate for the expansion of both outpatient and inpatient palliative care structures. The need for and access to palliative care treatment options for people with non-oncological diseases [54], [55] especially internal and neurological diseases, are particularly important. Although this has been discussed for several years [56], [57], [58], the results show that it is still perceived as insufficient. In Europe, the inadequacy of palliative care is also attributed to the lack of mandatory curricula in medical education [59].

### Strengths and weaknesses

The survey conducted at three medical faculties in North Rhine-Westphalia, allowing for a regional, although not nationwide, insight despite the small sample size. While all PY students at the three universities could be contacted, this was not feasible for all resident physicians, so their selection corresponded to a *purposive sample*.

These doctors at the beginning of their careers combine two perspectives: Their medical education is not far behind them, so they are still able to judge student education well. At the same time, as practicing physicians, they are in a position to make statements about knowledge transfer in clinical practice. To give particular weight to this dual perspective, they were given the opportunity to conduct in-depth interviews in addition to filling out the questionnaire, unlike the PY students. Overall, a bias with regard to the study population cannot be ruled out, as it can be assumed that the study participants had a general interest in palliative care content and were confronted with palliative care to varying degrees depending on their area of further training. In addition, it cannot be ruled out that the retrospective self-assessment of the resident physicians was influenced by the knowledge and competence gained during their clinical education. 

Regarding the PY students and their reported contact with palliative care, it should be noted that they had completed different tertials at the time of the survey and were working in departments with varying degrees of involvement in palliative care during their PY.

## Conclusions

The relevance of palliative care in clinical practice for the care of oncological and non-oncological patients requires practical palliative medicine education and courses that enables theory to be transferred into practice. This includes an intensification of practical small group teaching with, among other things, communication training with patients and their families, and involving students in the care of palliative patients, including therapy approach adjustment processes, already as students in order to strengthen expertise in dealing with this vulnerable group of patients and relatives in everyday life. A cross-university curriculum would help to lay a standardised foundation for palliative care education.

## Authors

### Shared authorship

The authors Marie-Christin Dronia and Kim Dillen share the first authorship.

### Authors’ ORCIDs

- Kim Dillen: [0000-0002-0270-3338]
- Frank Elsner: [0000-0001-5992-5591]
- Manuela Schallenburger: [0000-0002-3364-6137]
- Marin Neukirchen: [0000-0002-2287-7896]
- Anna Hagemeier: [0000-0002-8795-7352]
- Raymond Voltz: [0000-0002-4761-3395]
- Heidrun Golla: [0000-0002-4403-630X]

## Competing interests

The authors declare that they have no competing interests. 

## Supplementary Material

Online questionnaire for students in their practical year (PY)

Online questionnaire for resident physicians

Interview guideline (Interview guideline extended, IMEP-RU-Armenia)

Complete category system of the free-text responses from the questionnaires of the participating medical students during their practical year (PY) and resident physicians (coding frequency in brackets)

Category system of the interviews with coding frequencies

Quotes from the resident physicians

## Figures and Tables

**Table 1:**
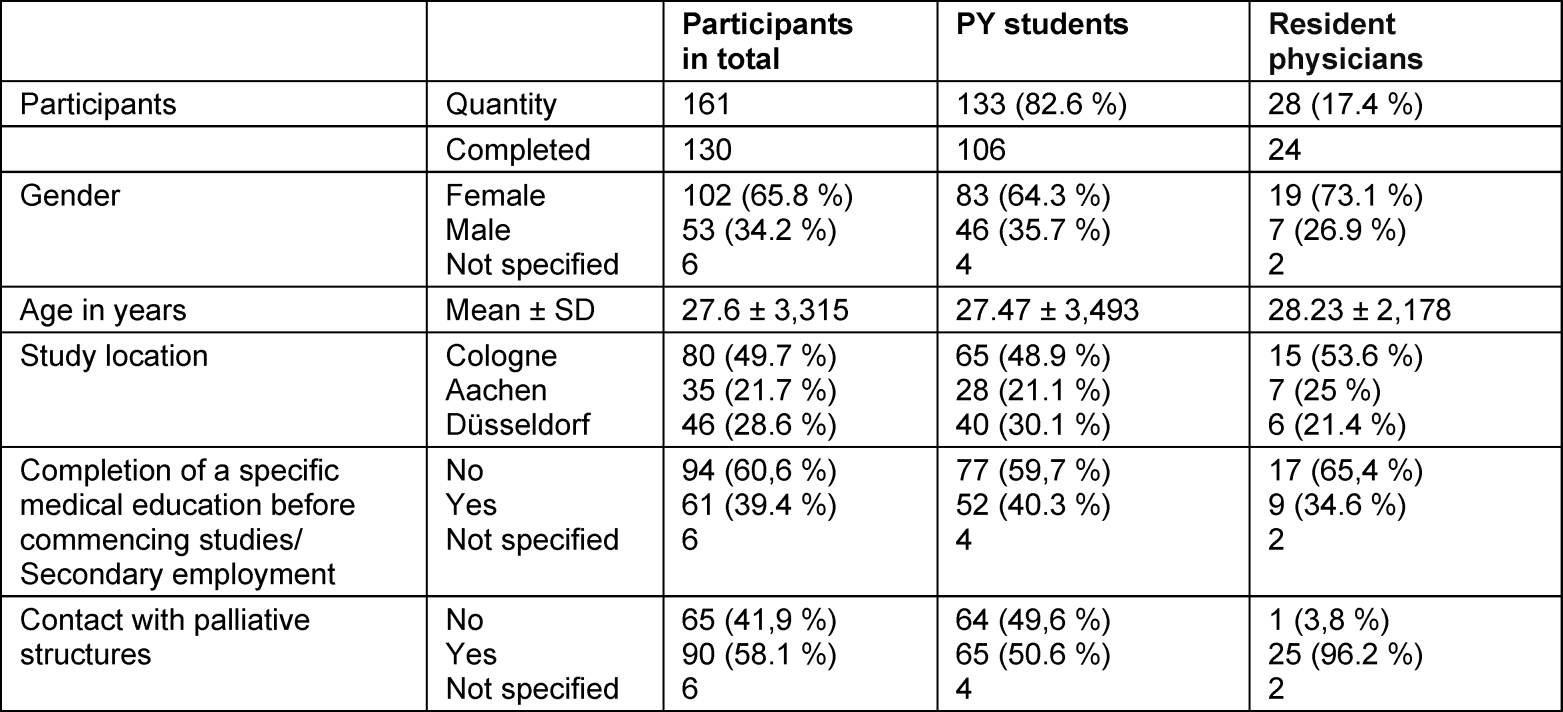
Participants

**Table 2:**
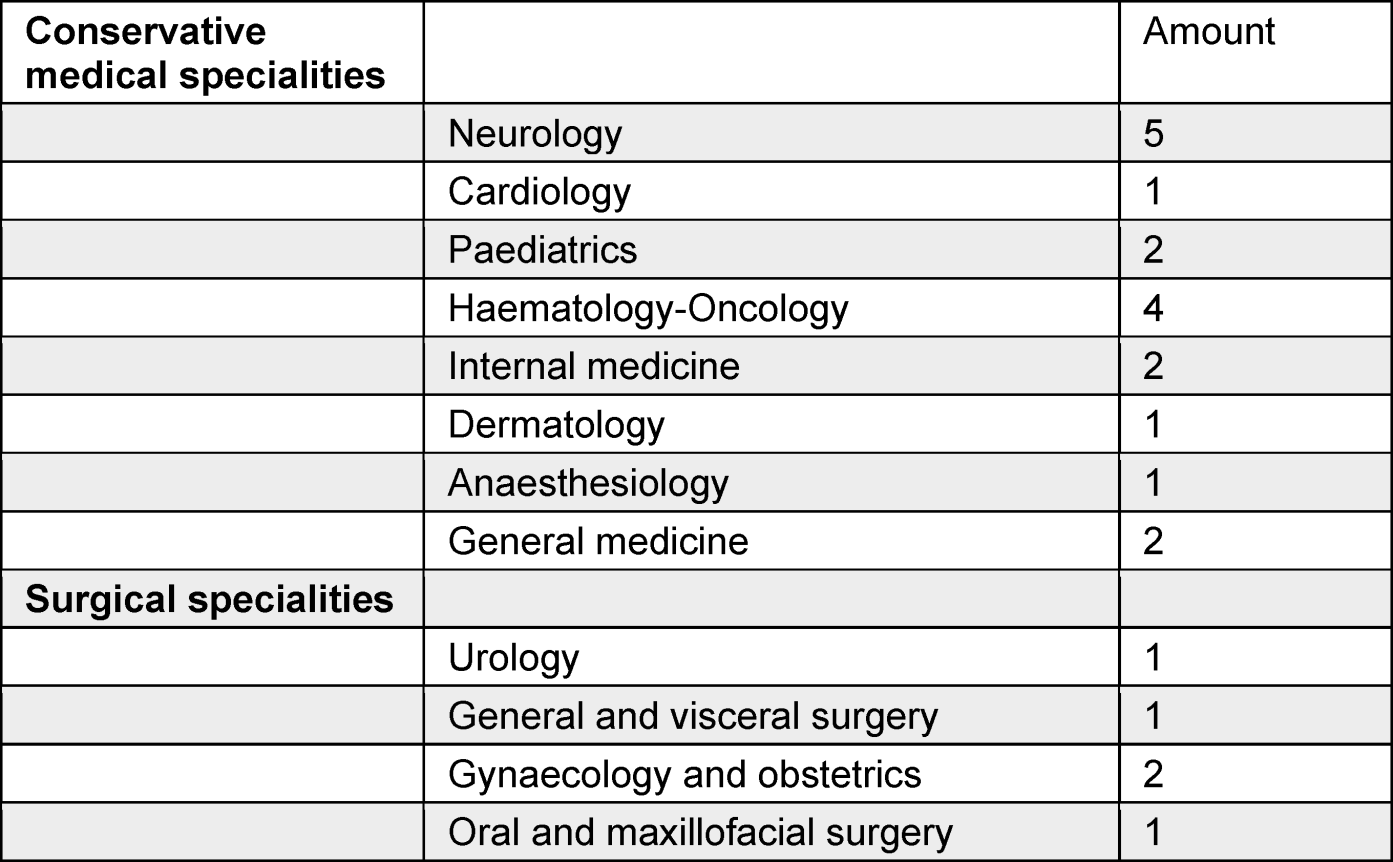
Current specialities of the residents physicians participating in the interviews (N=23)
