# Supplementary material for: Palliative care education and knowledge transfer into practice – a multicenter survey among medical students and resident physicians in Germany using a mixed-methods design
Source: GMS J Med Educ. 2024 Jun 17;41(3):Doc27. doi: 10.3205/zma001682 (PMC11310786; doi:10.3205/zma001682)
Supplement: Online questionnaire for students in their practical year (PY) [file JME-41-27-s-001.pdf]

Attachment 1: Online questionnaire for students in their practical year (PY)

Attachment 1 to Dronia MC, Dillen K, Elsner F, Schallenburger M, Neukirchen M, Hagemeier A, Hamacher S, Doll A, Voltz R, Golla H. *Palliative care education and knowledge transfer into practice – a multicenter survey among medical students and resident physicians in Germany using a mixed-methods design*. GMS J Med Educ. 2024;41(3):Doc27. DOI: 10.3205/zma001682

Structural data

Age: ______ Gender ☐ male ☐ female ☐ other
University: _________________
Have you already come into contact with palliative care structures (palliative care ward, doctors or nursing staff with further training in palliative care, palliative care service (PMD)[1], palliative outpatient clinic, palliative day clinic, specialised outpatient palliative care (SAPV), hospice (service), further training courses)?<br><br>☐ yes ☐ no<br><br>If yes, in which way?<br><br>…………………………………………………………………………
Did you complete a specific medical education before your studies or did you work in a medical institution during your studies?<br><br>☐ yes ☐ no<br><br>If yes, in which medical field did you work?<br><br>…………………………………………………………………………

What does palliative care mean to you?

…………………………………………………………………………………………………

…………………………………………………………………………………………………

…………………………………………………………………………………………………

How would you rate the proportion of palliative medicine teaching in the overall curriculum?

☐ too low ☐ just right ☐ too high

☐ not specified ☐ Other

Looking back, which palliative medicine course as part of QB13 prepared you best for dealing with patients with an incurable, advanced illness?

[1] Sometimes also referred to as a palliative medical consultation service

At the University of Cologne

☐ QB13 Lectures

☐ Competence Course: Death and mourning

☐ Competence Course: changing therapy goals

☐ Competence Course: Symptom control

☐ PY-START Course

☐ Internship in palliative medicine

☐ Practical Year tertial in palliative medicine

☐ Other events:

....

At the Heinrich Heine University Düsseldorf

☐ Palliative care Lectures

☐ Palliative care seminars

☐ Bedside teaching at the palliative care centre

☐ Internship in palliative medicine

☐ PY tertial in the palliative care centre

☐ Other events

....

At the RWTH Aachen

☐ Lectures

☐ Palliative care system block

☐ Seminar

☐ Traineeship in palliative medicine

☐ PY tertial in the palliative care centre

☐ Other events

....

Did you miss anything during your studies with regard to your palliative care training?

☐ yes ☐ no ☐ don't know

Which aspects may have been dealt with too briefly or not at all?

☐ Notification of serious diagnoses

☐ Dealing with patients in pre-final/final phases of life

☐ Change of treatment goal to a palliative concept

☐ Communication with relatives

☐ Symptom-relieving therapy (dyspnoea, pain, nausea, weakness...)

☐ Dealing with death and grief

☐ Other: ....

Were you involved in the communication of serious diagnoses during your practical year?

☐ Yes, I was often able to be part of these conversations

☐ I was rarely able to be present at these conversations

☐ No, these conversations were held without me

☐ Not specified

☐ Other

As a PY student, were you involved in the care and treatment of patients with a palliative care therapy concept?

☐ Yes, I was often able to care for patients with a palliative care concept under supervision

☐ Occasionally I was able to care for patients with a palliative care concept under supervision

☐ This opportunity never arose

☐ Not specified

Please rate your skills in relation to the following core palliative care competencies (from 1=strongly disagree to 5=strongly agree) [2]

**C 1**	**Core components of palliative care [3]**<br><br>I feel confident in naming the core components of palliative care.<br><br>1 ☐ 2 ☐ 3 ☐ 4 ☐ 5 ☐
**C 2**	**Physical well-being of palliative patients**<br><br>I can strengthen the physical well-being of patients during the course of their illness.<br><br>1 ☐ 2 ☐ 3 ☐ 4 ☐ 5 ☐<br><br>I feel confident in treating common symptoms that can occur in palliative care patients.<br><br>1 ☐ 2 ☐ 3 ☐ 4 ☐ 5 ☐<br><br>I feel confident in my knowledge of the causes of common symptoms that can occur in palliative care patients.<br><br>1 ☐ 2 ☐ 3 ☐ 4 ☐ 5 ☐<br><br>I feel confident in prescribing appropriate and adequate pain therapy.<br><br>1 ☐ 2 ☐ 3 ☐ 4 ☐ 5 ☐<br><br>I feel confident in my knowledge of the therapeutic effects and side effects of analgesics.<br><br>1 ☐ 2 ☐ 3 ☐ 4 ☐ 5 ☐<br><br>I feel confident in my knowledge of the therapeutic effects and side effects of symptom-relieving medication (other than analgesics).<br><br>1 ☐ 2 ☐ 3 ☐ 4 ☐ 5 ☐

[2] According to Marie Curie Palliative Care Institute Liverpool – SEPC & Thanatophobia Assessment Scale, German version – Clinic for Palliative Medicine, RWTH University Aachen

[3] Core components of palliative care: For example, understanding the importance of the physical, psychological, social and spiritual aspects of patients and their relatives, adopting a life-affirming attitude with a focus on quality of life, and the ability to recognise palliative care needs early enough.

**C 3**	**Dealing with psychological needs**<br><br>I feel able to assess the patient's needs.<br><br>1 ☐ 2 ☐ 3 ☐ 4 ☐ 5 ☐<br><br>I feel confident in being able to provide psychological care for palliative care patients.<br><br>1 ☐ 2 ☐ 3 ☐ 4 ☐ 5 ☐
**C 4**	**Dealing with social needs**<br><br>I feel confident in being able to provide social care for palliative care patients.<br><br>1 ☐ 2 ☐ 3 ☐ 4 ☐ 5 ☐
**C 5**	**Dealing with spiritual needs**<br><br>I feel confident in being able to provide spiritual care for palliative care patients.<br><br>1 ☐ 2 ☐ 3 ☐ 4 ☐ 5 ☐
**C 6**	**Care for relatives and dependants**<br><br>I feel able to assess the needs of the relatives of palliative care patients.<br><br>1 ☐ 2 ☐ 3 ☐ 4 ☐ 5 ☐<br><br>I feel confident in being able to provide psychological support to relatives of palliative care patients.<br><br>1 ☐ 2 ☐ 3 ☐ 4 ☐ 5 ☐<br><br>I feel confident in being able to provide social care for relativesof palliative care patients.<br><br>1 ☐ 2 ☐ 3 ☐ 4 ☐ 5 ☐<br><br>I feel confident in being able to provide spiritual care for relatives of palliative care patients.<br><br>1 ☐ 2 ☐ 3 ☐ 4 ☐ 5 ☐

**C 7**	**Ethical aspects**<br><br>I feel confident to include ethical aspects in the decision-making process.<br><br>1 ☐ 2 ☐ 3 ☐ 4 ☐ 5 ☐
**C 8**	**Care coordination and interdisciplinary teamwork**<br><br>I am able to work in an interdisciplinary and multi-professional team.<br><br>1 ☐ 2 ☐ 3 ☐ 4 ☐ 5 ☐<br><br>I feel confident in being able to involve different care centres and different professions in the treatment.<br><br>1 ☐ 2 ☐ 3 ☐ 4 ☐ 5 ☐
**C 9**	**Communicative skills**<br><br>I feel secure in myself talking to the patient about his/her death.<br><br>1 ☐ 2 ☐ 3 ☐ 4 ☐ 5 ☐<br><br>I feel confident in answering the patient's question "Will I suffer a lot or be in pain?"<br><br>1 ☐ 2 ☐ 3 ☐ 4 ☐ 5 ☐<br><br>I feel confident in answering the patient's question "How long do I have to live?"<br><br>1 ☐ 2 ☐ 3 ☐ 4 ☐ 5 ☐<br><br>I feel confident discussing the death of the patient after it has occurred.<br><br>1 ☐ 2 ☐ 3 ☐ 4 ☐ 5 ☐<br><br>I feel confident in discussing the (expected) death of the patient with the patient's relatives.<br><br>1 ☐ 2 ☐ 3 ☐ 4 ☐ 5 ☐
**C 10**	**Reflection and further training**<br><br>I am aware of the need for continuous reflection on my work.<br><br>1 ☐ 2 ☐ 3 ☐ 4 ☐ 5 ☐

Indicate the number whether you disagree or agree with the following statements (from 1=strongly disagree to 5= trongly agree). [4]

**C 11**	Dying patients give me a feeling of uncertainty.<br>1 ☐ 2 ☐ 3 ☐ 4 ☐ 5 ☐
**C 12**	I feel pretty helpless when I have dying patients on my ward.<br>1 ☐ 2 ☐ 3 ☐ 4 ☐ 5 ☐
**C 13**	It is frustrating to have further conversations with relatives of patients who cannot expect a recovery.<br>1 ☐ 2 ☐ 3 ☐ 4 ☐ 5 ☐
**C 14**	Caring for dying patients traumatises me.<br>1 ☐ 2 ☐ 3 ☐ 4 ☐ 5 ☐
**C 15**	I feel uncomfortable when a dying patient wants to say goodbye to me.<br>1 ☐ 2 ☐ 3 ☐ 4 ☐ 5 ☐
**C 16**	I do not look forward to being the doctor caring for a dying patient.<br>1 ☐ 2 ☐ 3 ☐ 4 ☐ 5 ☐
**C 17**	When patients start talking about death, it makes me uncomfortable.<br>1 ☐ 2 ☐ 3 ☐ 4 ☐ 5 ☐

D How do you determine the palliative care needs of a patient?

- ☐ Type of illness
- ☐ Stage of the disease
- ☐ Symptom burden
- ☐ Patient request
- ☐ Other: ___________________

[4] From Marie Curie Palliative Care Institute Liverpool – SEPC & Thanatophobia Assessment Scale, German version – Clinic for Palliative Medicine, RWTH University Aachen

In your opinion, when is the right time to integrate palliative medical treatment?

………………………………………………………………………………………………….

………………………………………………………………………………………………….

…………………………………………………………………………………………………..

…………………………………………………………………………………………………..

In your opinion, which non-oncological diseases require palliative care?

☐ ALS

☐ M. Parkinson

☐ COPD

☐ Heart failure

☐ Renal insufficiency

☐ Other: _________________

How do you rate palliative medical care for patients in an inpatient setting? Please select one of the following answers:

☐ Completely sufficient

☐ Mostly sufficient

☐ Rarely sufficient

☐ Not sufficient

☐ Other: _________________

☐ Not specified

It is reasonable to set up a separate palliative care ward in hospitals/clinics.

☐ Agree ☐ Do not agree ☐ Don't know

It is reasonable to set up a palliative care service in hospitals/clinics.

☐ Agree ☐ Do not agree ☐ Don't know

Outpatient palliative care services, e.g. specialised outpatient palliative care (SAPV), are a useful addition to the services provided by general practitioners/specialists.

☐ Agree ☐ Do not agree ☐ Don't know

The following lines are intended to give you the opportunity to make remarks or comments independently of the questions we have asked. Perhaps you can remember an example of a patient or a situation that had a particular impact on you.

....

....

....

....
