# Supplementary material for: Palliative care education and knowledge transfer into practice – a multicenter survey among medical students and resident physicians in Germany using a mixed-methods design
Source: GMS J Med Educ. 2024 Jun 17;41(3):Doc27. doi: 10.3205/zma001682 (PMC11310786; doi:10.3205/zma001682)
Supplement: Interview guideline (Interview guideline extended, IMEP-RU-Armenia) [file JME-41-27-s-003.pdf]

Attachment 3: Interview guideline (Interview guideline extended, IMEP-RU-Armenia)

First of all I would like to thank you very much on agreeing to answer a few questions for me. It will take approximately 30 to 45 min. for the whole interview. Is that fine for you?
Remember all that you will say here will be kept confidentially.
You have already been told that we developed this study for a better understanding of how
Doctors feel prepared to deal with the challenges in caring for incurable and dying patients.

**1. Professional development**	**Could you please give me a brief summary of your professional development so far?**<br>- Education?<br>- University?<br>- Department?
**2. Personal definition of palliative care**	**Please could you describe your personal understanding of palliative care?**<br>- What would be your own definition of this subject?<br>- In which way do you think is Palliative Care different to the treatment of other Departments?<br>- Do you know if there is Palliative Care in Armenia?<br>- Do you have any experience with palliative care? So besides your job?
**3. Education**	**Could you describe your experience of education in palliative care?**<br>- At the university / lectures / workshops/ anything voluntary?<br>- At your department / training for residents?<br>- What have they taught you?

**4. Quality of education**	**Could you tell us about how you experience the quality of the education in palliative care?**<br>- Do you feel more prepared for the care of incurable and dying patients and their families after the education?<br>- Has this affected your ability to provide care +/-
**5. Communication**	**How confident and able do you feel to**<br>- deal with difficult questions<br>- tell patients / families that they<br>• cannot be cured / are dying?<br>• Do you use the word death in conversation with the family?<br>**Is it more common in Armenia to inform the patient about his medical situation or his/her relatives/family instead?**<br>- Where have you learnt to handle situation like that?<br>- In your opinion: what do you think is the right way? Informing the patient or the family/relatives?
**6. Pain and Symptom Management**	**How confident and able do you feel to provide Pain and Symptom Management in a palliative situation?**<br>- What does it mean to you?
**7. Diagnosing Dying**	**How confident and able do you feel to diagnose dying?**<br>- Have you learnt something about it?<br>- Have you learnt how to tell the patient/family?<br>- Is it common in Armenia to ask for a prognosis? What do you think about it?
**8. Working in a Multidisciplinary Team**	**What does it exactly mean to you?**<br>- Which departments do you think includes the team?
**9. Attitudes to Dying and Death**	**Could you describe your attitudes towards death and dying?**<br>- As a Health Care Professional (attitudes towards caring for a dying patient and their family)<br>- As an individual (what it means to the person being interviewed)

**10. The personal support from faculty received by the doctors**	**Could you describe the personal support from the faculty you did receive?**<br>- What kind of support?<br>- Who supported you? Department? Attendant? Psychologist?
**11. Role concept**	**What is your idea of your role as a physician?**<br>- How do you see yourself as a doctor?<br>(save lives/ getting patients cured/help them to a better health/ make their illness as acceptable/comfortable as possible)
**12. At least: The future development of palliative care**	**What are your hopes regarding future palliative care?**<br>- Would it be helpful to have more education in this subject?

Thank you very much you have contributed to a better understanding of how young doctors feel prepared to deal with the challenges of caring for incurable or dying patients.

Do you have any further questions?

Attachment 3 to Dronia MC, Dillen K, Elsner F, Schallenburger M, Neukirchen M, Hagemeier A, Hamacher S, Doll A, Voltz R, Golla H. *Palliative care education and knowledge transfer into practice – a multicenter survey among medical students and resident physicians in Germany using a mixed-methods design*. GMS J Med Educ. 2024;41(3):Doc27. DOI: 10.3205/zma001682
