# Supplementary material for: Palliative care education and knowledge transfer into practice – a multicenter survey among medical students and resident physicians in Germany using a mixed-methods design
Source: GMS J Med Educ. 2024 Jun 17;41(3):Doc27. doi: 10.3205/zma001682 (PMC11310786; doi:10.3205/zma001682)
Supplement: Complete category system of the free-text responses from the questionnaires of the participating medical students during their practical year (PY) and resident physicians (coding frequency in brackets) [file JME-41-27-s-004.pdf]

Attachment 4: Complete category system of the free-text responses from the questionnaires of the participating medical students during their practical year (PY) and resident physicians (coding frequency in brackets)

Main category	Subcategories	Sample quotes (questionnaire number)
Contact with palliative care structures	Palliative care consultation service (N=30)	“Regularly in everyday clinical practice with the palliative care consultation service” (FB 514768-12)
	SAPV organisation (N=11)	“SAPV planning” (FB 514768-14)<br>“Outpatient care (SAPV)” (FB 519847-40)
	As part of inpatient care (N=27)	“Inpatient care of oncological patients -> close cooperation with colleagues in palliative medicine/palliative care-trained staff” (FB 514768-8)<br>“As part of the nursing activities (palliative care ward, consultation service, etc.)” (FB 432212-63)
	MVZ Palliative Medicine (N=3)	“MVZ Palliative Medicine CIO” (514768-10)
	Transfer, palliative care unit, hospice (N=14)	“Transfer to the palliative care unit/hospice” (FB 392169-3)
	In Courses/PY/clinical traineeship (N=40)	“Participation in the qualification profile “the patient as teacher” during the degree programme” (FB 392169-2)<br>“Clinical traineeship 2 weeks on a palliative care ward” (FB 392169-6)
	Personal experience/private (N=9)	“Volunteering in the hospice” (FB 432212-27)
Training completed before/during studies or part-time employment in a medical institution	Sleep laboratory (N=6)	“Sleep laboratory” (FB 519847-4)
	SHK (N=24)	“Student assistant in neurology” (FB 514768-5)<br>“SHK GP practice” (FB 432212-38)
	Activity in healthcare and nursing (N=19)	“Inpatient care in a hospital (night shift) as a student assistant” (FB 514768-3)<br>“Intensive care nurse” (FB 432212-2)
	Blood collection aid (N=6)	“As a blood collection student during my studies on the internal medicine wards” (FB 392169-7)
	Emergency room/ambulance service (N=6)	“Rescue service” (FB 432212-8)<br>“Emergency room care” (432212-15)
	Laboratory/research (N=5)	“Laboratory/ Pathology as MTLA” (FB 432212-12)<br>“In research” (FB 432212-50)
	Pharmacology (N=1)	“Pharma (CRO)” (FB 432212-14)
	Health authority (N=1)	“In the health department” (FB 432212-27)
	Outpatient sector (N=4)	“In (a) medical practice as a medical assistant” (FB 432212-27)

Attachment 4 to Dronia MC, Dillen K, Elsner F, Schallenburger M, Neukirchen M, Hagemeier A, Hamacher S, Doll A, Voltz R, Golla H. *Palliative care education and knowledge transfer into practice – a multicenter survey among medical students and resident physicians in Germany using a mixed-methods design*. GMS J Med Educ. 2024;41(3):Doc27. DOI: 10.3205/zma001682

Question: What does palliative care mean to you?	Treatment of incurable diseases/non-curative therapy approach (N=85)	“The medical care of patients who are no longer pursuing curative therapy.” (FB 514768-13)
	Accompanying relatives (N=24)	“Holistic support for patients and relatives.” (FB 514768-4)<br>“Palliative care is not just symptom-controlled support for patients, but also a place of trust for their relatives. They don’t feel alone and know that they don’t have to go through this suffering alone.” (FB 432212-28)
	Holistic, individualised therapy approach (N=41)	“Holistic support for patients and relatives.” (FB 514768-4)<br>“Individualised medicine with the aim of maximising quality of life.” (FB 392169-3)
	Part of general care (N=6)	“Essential part of everyday patient care.” (FB 514768-8)
	Medical/medical measures (N=10)	“No curative therapy approach, pain control, final phase of life.” (FB 563111-4)<br>“No cure to be expected, end-of-life care, symptom control” (FB 563111-22)
	Non-medical/non-medical measures (N=17)	“Improvement in quality of life through symptom control, psychological support.” (FB 392169-6)<br>“The medical care of patients who are no longer pursuing curative therapy. In particular, the focus is on alleviating suffering with the help of medicinal and non-medicinal treatments.” (FB 514768-13)
	Symptom control/relief, symptom-orientated (N=55)	"“ased on the concept of not striving for a cure but for symptom relief.” (FB 514768-2)<br>“Quality of life instead of lifetime, focussing on symptoms and life circumstances.” (FB 519847-18)
	Quality of life and dignity (N=63)	“Maintaining or improving the quality of life at the end of life.” (FB 514768-15)<br>“Dignified care for life and death.” (FB 475915-1)
	End-of-life care + assistance (N=46)	“Furthermore, an emotional “preparation”/confrontation with the patients for a possible dying process.” (FB 392169-3)<br>“Organising and accompanying death with dignity.” (FB 432212-10)

	Relief from suffering and anxiety (N=10)	“Give the days more life, avoid unnecessary agony.” (FB 514768-10)<br>“An important branch of medicine for alleviating suffering and maintaining the best possible quality of life in the case of incurable, advanced diseases.” (FB 432212-4)
	(Organisation of) outpatient care (N=6)	“No more cure, focus on symptoms, inpatient and outpatient care to avoid hospitalisation.” (FB 432212-65)<br>“Optimisation of care at home.” (FB 519847-18)
	Counterpoint to curative departments (N=3)	“Focus on the real, current needs of patients and support during serious illness as opposed to diagnosis- and prognosis-based medicine.” (FB 392169-5)<br>“Alternative treatment goal to curative medicine.” (FB 432212-14)
	Communication (N=1)	“The decision to switch to a palliative concept, empathetic communication with the patient and relatives, patient support and symptom-relieving therapy to support the patient in the last phase of life and minimise painful experiences.” (FB 392169-7)
	Change in therapy goal (N = 1)	“Change of therapy goal” (FB 432212-39)
Question: In your opinion, when is the right time to integrate palliative medical treatment?	Change in treatment goal, no longer curative approach, focus on quality of life (N=60)	“If a cure is not foreseeable and a change in treatment goal occurs, if care is to be guaranteed in the home in the event of persistent poor health.” (FB 514768-2)
	Discussion of therapy goal changes/meaningful measures (N=6)	“If there are uncertainties regarding a possible change of treatment goal or if this decision has been made by us (at the latest then).” (FB 514768-1)<br>“After a change in treatment goal, before decisions regarding a palliative concept, in difficult situations (symptom burden, environment/relatives).” (FB 432212-62)
	Organisation of home care (N=2)	“If a cure is not foreseeable and a change in treatment goal occurs, if care is to be guaranteed in the home in the event of persistent poor health.” (FB514768-2)
	Individual (N=9)	“To be determined individually, depending on the patient's well-being and wishes.” (FB 519847-32)<br>“Very different depending on the disease and symptoms, but also depending on age and previous illnesses.” (FB 514768-3)

	Patient request (N=17)	“If the patient so wishes. If the prognosis is very limited and the patient has been informed, but may not yet have entered the process of acceptance. If there are severe symptoms that can be supported with supportive therapy by the PAL Consultation Service.“ (FB 514768-9)<br>“If required for support, at the request of patients.” (FB 519847-16)
	Demand-orientated (N=60)	“If further measures take away more quality of life than they give, psychological and social care is needed, uncontrollable symptoms may occur in the future, symptoms or diagnosis indicate a terminal stage.” (FB 432212-9)<br>“Regardless of disease and stage, if there is a need.” (FB 432212-50)
	As early as possible/at diagnosis (N=38)	“As early as possible. So as soon as the patient is given a diagnosis that is likely to be terminal, he should, in my opinion, be offered palliative medical counselling.“(FB 392169-1)<br>“When an incurable, advanced disease is diagnosed.” (FB 432212-4)
	For symptom control even under potentially curative therapy (N=8)	“Early enough, even for symptom control during therapy.” (FB 514768-11)
	Wish of the relatives (N=3)	“if there is a need on the part of patients and relatives” (FB 563111-4)
	If need is recognised by doctor (N=1)	“if doctor or patient see a need” (FB 563111-27)

Attachment 4 to Dronia MC, Dillen K, Elsner F, Schallenburger M, Neukirchen M, Hagemeier A, Hamacher S, Doll A, Voltz R, Golla H. *Palliative care education and knowledge transfer into practice – a multicenter survey among medical students and resident physicians in Germany using a mixed-methods design*. GMS J Med Educ. 2024;41(3):Doc27. DOI: 10.3205/zma001682
