# Supplementary material for: Palliative care education and knowledge transfer into practice – a multicenter survey among medical students and resident physicians in Germany using a mixed-methods design
Source: GMS J Med Educ. 2024 Jun 17;41(3):Doc27. doi: 10.3205/zma001682 (PMC11310786; doi:10.3205/zma001682)
Supplement: Category system of the interviews with coding frequencies [file JME-41-27-s-005.pdf]

Attachment 5: Category system of the interviews with coding frequencies

Code name	Quantity
Definition of palliative care	
- Early integration	1
- WHO definition	1
- Multidisciplinary	2
- End-of-life care	2
- Quality of life	13
- Differences to other specialised departments	4
- Not only end-of-life care	3
- Organisation of outpatient care	3
- Holistic, individualised	15
- Change of therapy goal	5
- Specialised team	14
- Involvement of relatives	8
- Symptom control	16
- Patients with a final diagnosis, no curative approach	14
- Not only oncological diseases with a need	3
Experience with palliative care	
- Private	2
- Research	1
- PY Palliative Medicine	4
- Internship in palliative medicine	3
- Presence/lobby not given everywhere	2
- Theoretical contact	1
- Different frequency of contact	4
- Integration of palliative care services	15
Attitude towards dying and death as a doctor or individual/private person	
- No/little difference	7
- Differences in coping	5
- Spirituality	4
Attitude towards dying and death as a doctor	
- Pragmatic approach	20
- Integrity	2
- Awareness that patients can die	7
- Lack of awareness of dying/death	1
- avoid dying	4
- Meaningfulness of action is questioned	5
- Depending on the situation	4
- Acceptance difficult	3
Attitude towards dying and death as an individual/private person	
- Emotional component	9
- Rationality	3
- No fear of death	3
- No/few points of contact	2
- Ignorance	2
- Dealing with death, Patient decree	8

Attachment 5 to Dronia MC, Dillen K, Elsner F, Schallenburger M, Neukirchen M, Hagemeier A, Hamacher S, Doll A, Voltz R, Golla H. *Palliative care education and knowledge transfer into practice – a multicenter survey among medical students and resident physicians in Germany using a mixed-methods design*. GMS J Med Educ. 2024;41(3):Doc27. DOI: 10.3205/zma001682

Teaching	
Teaching: Courses/content mentioned	
- E-learning	3
- Practical course palliative care	3
- Elective Courses<br><br>→ Experience course borderline situations<br>→ The patient as teacher<br>→ Communication with the dying	5<br><br>2<br>2<br>1
- Lectures	9
- KF Change of therapy goal	4
- Grief model	1
- Communication training	17
- Practical lessons/small group sessions	12
- Symptomcontrol	3
- KF Death and mourning	2
- Multiprofessional teachers	1
Teaching: Influence of teaching on the care skills of palliative care patients	
- Importance of Practical Year	4
Teaching: Influence of teaching on the care capabilities of palliative care patients:<br>Positive aspects	
- Teaching extensive, nothing missing	2
- Small group lessons/seminars	1
- Skills/basic attitude learnt	22
- Communicative skills learnt	5
- Changes in therapeutic goals, mediated dying	1
- Larger focus on palliative care education in Germany than abroad	1
- Patient contact/practical relevance	4
- Outpatient offers	1
Teaching: Influence of teaching on the care-related skills for palliative care patients:<br><br>Negative aspects	
- Only "compulsory seminar"	2
- Repetitions	1
- Transfer in everyday clinical life difficult	7
- Too little clinical reference	2
Teaching: Influence of teaching on the care-related skills for palliative care patients:<br>Problems	
- Fear of contact	1
- Not sufficient	5
- Implement changes in therapy goals	4
- Lack of time in patient care	3
- No therapy, pure support	1
- Everyday clinical practice is different from the illustration in the courses	4

Teaching: Influence of teaching on the care-related skills for palliative care patients:<br>Communication	
- Learned from others in the clinic	2
- Communication training has had a positive effect	13
- Content from communication training is still present	12
- Independent further training	1
- Conversations with relatives more difficult	1
Teaching: Influence of teaching on the care-related skills for palliative care patients:<br>Suggestions for improvement	
- Convey basic knowledge/attitude	2
- Breaking up seminar structures	1
- Expansion of teaching/patient contact	2
- Practical instruction/application	7
- Working methods/advantages of palliative care structures	1
- Communication<br>→ Expand dialogue training<br>→ Communication with relatives	5<br>4<br>1
- Symptom control (including implementation, dosage)	6
Teaching: Influence of teaching on the care-related skills for palliative care patients:<br>Dying as a topic in university teaching	
- Has been treated	1
- Only in palliative care	3
- Little access even in clinics	2
- Learning from other professions	1
- Difficult realisation	5
- Too little thematised	15
- Is learnt "clinically"	19
- Was not thematised	8

**Core competencies in palliative medicine<br>Symptom control**	
Involvement of other services/professions	14
- SAPV	2
- Palliative care service	8
- Care	3
- Palliative care unit	1
- Other specialised departments	1
Theoretical concept development	3
Assessment of the ability to provide adequate symptom control:<br>Problems	25<br><br>13
- Outpatient care	2
- Difficult realisation in everyday life	2
- Control of rare/specialised symptoms<br>→ Phytotherapy<br>→ Psychological symptoms, anxiety<br>→ Malnutrition<br>→ Constipation<br>→ Delirium	7<br>1<br>3<br>1<br>1<br>1
- Non-drug therapy	2
- Pain therapy	6
- Holistic concept	3
- Skills dependent on patient/situation	3
- Still need to learn/need support	8
- Dosage/clinical implementation	6
Symptom check: Assessment of the ability to provide adequate symptom control:<br>Expertise	 <br>13
- Common symptoms well manageable<br>→ Loss of appetite<br>→ Pre-final/final phase<br>→ Pain/pain medication<br>→ Nausea/vomiting<br>→ Dyspnoea<br>→ Fear	10<br>1<br>4<br>10<br>9<br>5<br>1
- Good foundation/entry through apprenticeship	5
**Core competencies in palliative medicine<br>Communication**	
Communication: Dealing with the word "death"	20
- Avoiding "death"/difficulties	6
- Conscious, clear address, specific naming	21
- Paraphrasing<br>→ Dying/dying is preferred to “death”	15<br>(5)
- Depending on the patient/conversation situation	6
- So far only observed, conversations not conducted	7

Attachment 5 to Dronia MC, Dillen K, Elsner F, Schallenburger M, Neukirchen M, Hagemeier A, Hamacher S, Doll A, Voltz R, Golla H. *Palliative care education and knowledge transfer into practice – a multicenter survey among medical students and resident physicians in Germany using a mixed-methods design*. GMS J Med Educ. 2024;41(3):Doc27. DOI: 10.3205/zma001682

Communication: Communication with family members	11
- Clear/targeted communication	7
- Grief work	1
- Difficulties/problems<br>→ Unpleasant situations<br>→ Deliberately not included<br>→ Rejection of palliative connection<br>→ No insight<br>→ You want to escape the situation<br>→ Corona<br>→ Dealing with relatives more difficult than with patients<br>→ Frustration	12<br>2<br>1<br>1<br>1<br>2<br>3<br>1<br><br>1
Communication: Assessment of communication skills for difficult questions<br>Skills/abilities	
- Confidence in dealing with difficult issues	13
- Orientation/rel. safety through teaching	7
- Show empathy/understanding	3
- Learning-by-doing	5
- Sense of ability depending on the situation	3
Skills/abilities: Forecasts	21
- In which framework<br>→ OP clarification<br>→ Within the scope of studies<br>→ Asked by patients<br>→ Asked by relatives/associates	12<br>1<br>1<br>5<br>7
- Frequency<br>→ Often asked<br>→ Not a frequent question	14<br>12<br>2
- Handling<br>→ Clear communication<br>→ Time of the conversation<br>→ Lifetime statements<br>→ Difficult question<br>→ Cautious response<br>→ Orientation towards superiors	 2<br>3<br>15<br>8<br>10<br>1
Communication: Assessment of communication skills for difficult questions<br>Problems	17
- Conversations not a task for the assistants	5
- No support/supervision	2
- Lack of time as a doctor	2
- Enduring difficult situations	1
- Still need to learn	7
- Conversation difficult	4
- Difficult to provide spiritual support	2
- Uncertainty/emotionality in conversation	4

**Work in a multidisciplinary team**	23
Definition: Participants	
- Cleaning staff	1
- Radiotherapists	1
- Psychologists, counsellors	10
- Palliative medicine	9
- Curative specialised disciplines	11
- Social service	6
- Pain medicine	2
- Healthcare sector (physiotherapy, occupational therapy, speech therapy, etc.)	11
- Patient/relatives	1
- Nurses	6
Definition: Tasks	
- Exchange/feedback/reflection	11
- Contributing expertise	12
- Determine procedure together	3
Valuation	
- Bi-professional in everyday life	1
- Sometimes difficult to implement in practice	4
- Very helpful/appreciation/profit	17
- Good handling of complex cases	4
- Explicit evaluation PMK	6
- Expansion makes sense	8
- No implementation/individual decision-makers in day-to-day inpatient work	3

Understanding of roles	
- Human	1
- Actors, adapted to the situation	1
- Helper/companion	2
- Service provider	2
- Accepting the patient's wishes	2
- Improve living situation/suffering	4
- Supporter, motivator	1
- Holistic practitioner	4
- Healing, curative approach	4
- Therapist, counsellor	4
- Informant, contact person	10
- Practitioner of physical conditions	6

Attachment 5 to Dronia MC, Dillen K, Elsner F, Schallenburger M, Neukirchen M, Hagemeier A, Hamacher S, Doll A, Voltz R, Golla H. *Palliative care education and knowledge transfer into practice – a multicenter survey among medical students and resident physicians in Germany using a mixed-methods design*. GMS J Med Educ. 2024;41(3):Doc27. DOI: 10.3205/zma001682

Hopes and wishes for the future development of palliative care in Germany	
- Palliative care as an independent speciality	1
- Acceptance/implementation/no competition on the palliative care ward	9
- Strengthening the department within teaching	11
- No purely oncological focus	4
- Holistic treatment of all patients	2
- Pursue individual therapy approaches	1
- Public relations/lobbying	7
- Access/transfer	26

Support in difficult situations	
- Contact within the clinic<br><br>→ Supervision<br>→ Hospital chaplains, psychologists<br>→ Palliative physician<br>→ Psycho-oncology	 <br>3<br>6<br>1<br>1
- Case discussions	4
- Support from colleagues/doctors	27
- Distancing	1
- Private resources	10
- Desire for support	10
Support in difficult situations<br>Problems/challenges	
- Problems opening up to colleagues/doctors	4
- Emotional stress	3
- Clinic offer not yet utilised	2
- Support must be actively sought, not a matter of course	5
- Taking responsibility as a team	2
- Too little/no space	4
- Not planned/desired	9
- No support from superiors	4
- No known contact	12

Attachment 5 to Dronia MC, Dillen K, Elsner F, Schallenburger M, Neukirchen M, Hagemeier A, Hamacher S, Doll A, Voltz R, Golla H. *Palliative care education and knowledge transfer into practice – a multicenter survey among medical students and resident physicians in Germany using a mixed-methods design*. GMS J Med Educ. 2024;41(3):Doc27. DOI: 10.3205/zma001682
