# Supplementary material for: Palliative care education and knowledge transfer into practice – a multicenter survey among medical students and resident physicians in Germany using a mixed-methods design
Source: GMS J Med Educ. 2024 Jun 17;41(3):Doc27. doi: 10.3205/zma001682 (PMC11310786; doi:10.3205/zma001682)
Supplement: Quotes from the resident physicians [file JME-41-27-s-006.pdf]

Attachment 6: Quotes from the resident physicians

Original German quotes, translated into English

Topic	Quote number	Quote
**Palliative care in everyday clinical practice**	1	I worked on the ward for about a year and a half and had quite a lot of contact with the palliative care consultation service and ultimately also discussed these issues with the patients (...) and we actually had a very good relationship with the consultation service, with the colleagues from the palliative care consultation service (...). (D03, item 3)
	2	I really, really enjoy working with the palliative care doctors. (A02, item 35)
	3	(...) and it is always very pleasant to work with the palliative care physicians from the consultation service. (K06, item. 6)
	4	And I also find it very difficult when it comes to spiritual support and I think it's good that there are other people to turn to. (K04,item 24)
**Evaluation of teaching in QB 13**<br>*Type of events*	5	But what I remember is that it was always a lot of small group lessons. Much more than in other departments, you always had the feeling that a lot of effort was made and that it was very important. And that you have already learnt a lot through small group lessons and also through a lot of participation and have been able to discuss a lot. (K03, item 9)
	6	I know that we had simulated patients with whom we conducted practice interviews. How do I deliver the diagnosis of a terminal illness or how do I deal with the fact that I have to break the news of death to relatives? That we had acting patients or members of the acting profession with whom we acted it out and a supervisor who then explained to us afterwards how the whole thing works properly, how to organise such a conversation or, what also stuck with us, how to structure such a conversation in general. (A01, item 9)
**Evaluation of teaching in QB 13**<br>*Evaluation of the transfer performance*	7	We also had these small group lessons and we also had this seminar, which I thought was really good because you could apply what was said in the lecture to case studies. I can simply say that it was like this in the lecture: Okay, these are your tools of the trade. And in the seminars: Okay, how can you use it? How can we make this particular situation better? What can you offer the patient? And I think that here in Cologne we have always somehow made it clear that there are palliative physicians. (K10, item 7)
	8	I already have the feeling that teaching palliative medicine has influenced my skills. Well, that was actually one of the good study blocks. I really think that you've already learnt a lot because, above all, you had no connection to it before. Not even as a student. And I think you have learnt quite a lot. And now, what medication do I give if he has this and that, but also a bit of social welfare care. (D01, item 14)
	9	And, of course, in palliative care it also has a lot to do with personality, whether you can do it or not. But I think the medical aspect alone, the teaching definitely helped. You know that I do this when I'm short of breath and my pain is like this, and I dare to give MSI sometimes, even if the respiratory drive then becomes less, but that's exactly what we want. These are contents that were also well communicated. And, of course, I always saw that in my final year and so it was a bit easier for me to implement on the ward. (A06, item 9)

Attachment 6 to Dronia MC, Dillen K, Elsner F, Schallenburger M, Neukirchen M, Hagemeier A, Hamacher S, Doll A, Voltz R, Golla H. *Palliative care education and knowledge transfer into practice – a multicenter survey among medical students and resident physicians in Germany using a mixed-methods design*. GMS J Med Educ. 2024;41(3):Doc27. DOI: 10.3205/zma001682

**Evaluation of teaching in QB 13**<br>*Reducing fears and inhibitions*	10	What definitely helped was that it was made clear in the seminars that there can and should be acceptance of dying. And that this is part of the change in therapy goals, that you accept dying. Even if this may go against the intuitive desire to do something curative after all. That this is simply okay and that it is accepted. And with this basic attitude, that you were primed for it a bit and that you were made aware of it a bit. This helps you to accept it better in everyday life and to realise that it is simply okay to pursue other therapeutic goals. (K05, item 11)
	11	And it's absolutely important that you have it as part of your studies, but I can't complain about that now because we already learnt about it at university and had contact with it before and also did a compulsory internship there for a week. So that you also lose your fear of contact a little. I also think that's important for the students, but that's already the case here. (D02, item 34)
	12	So I think what I learnt in the qualification profile, which was a voluntary but additional apprenticeship, was definitely that. Not just in terms of content, but also that you perhaps lose your inhibitions a little or lose the fear that you can do something wrong and that you have already gained an insight. (A03, item 10)
**Evaluation of teaching in QB 13 Palliative Medicine**<br>*Problems with concrete implementation in everyday life*	13	I definitely felt better prepared than if we hadn't had it, but it was still nowhere near enough. So in the first situations where you had these situations as a young junior doctor, where you had to have these conversations or were confronted with these patients, it was an emotional burden in itself and you had to get used to such conversations first. So, how do I actually conduct this now, and then develop my own style of dialogue first. (A01, item 11)
	14	I really felt very helpless at the beginning when I had my first patient who needed a morphine perfusor, for example. I had absolutely no idea how to set up this perfusor, what I should do, how I should arrange it. (K 08, item 20).
**Evaluation of teaching in QB 13 +**<br>*Problems with concrete implementation in everyday life - symptom control*	15	I feel relatively safe when I'm in pain. I will say that I have experience in prescribing anaesthetics and I have no inhibitions about prescribing them for patients who are in a palliative treatment situation or in a metastasised treatment situation. I also have BTM prescriptions and write them myself. I have no problem with that. (D03, item 16)
	16	In the case of nausea, we have already discussed this quite well, i.e. what the main symptoms are and what the appropriate medication is. So, I know what I need or should take and would then have to deal with the dosage again. (D04, item 22)
	17	Yes, I would say partly. There is a wide range, which we also experience, be it anxiety, shortness of breath, a feeling of dryness or nausea. So I would say that I can actually cope well with things like that. (K01, item 20)
	18	Or also – we now have very few of these on the ward – but perhaps also things that are not treated with medication. So it's not that you're just handing over projections, but that you can use communicative strategies to alleviate symptoms such as anxiety. Or even alleviate anxiety with medication, without wanting to use excessive means with the patient. But that you shield yourself a little. So these psychotropic drugs, I'm just saying in the broadest sense, I'm not that sharp on that now. (A03, item 19)
	19	I just haven't had that so often. (...) So severe anxiety would be more difficult for me, I think (A05, item 18)

	20	Other symptoms that are more difficult to control, but which perhaps cannot be formulated in such general terms. So, for example, patients who are simply (...) for example, malnourished, I'll just call it that, in the last part of their lives. This is now something that is simply very difficult to handle. And yes, for example, nutrition in general. And then there are a few, I'll call them minor – but they shouldn't be less important – symptoms such as obstipation or, in very terminal patients, delirium, delirious experiences. (06 C, item 17)
**Evaluation of teaching in QB 13**<br>*Problems with concrete implementation in everyday life – change in therapy goal*	21	So, what was really the big difficulty for me at the beginning was to really make the diagnosis, i.e. to really draw the line between when therapeutic measures are enough and when it's just symptom control and pain control. So that's the limit, but that's probably also difficult and differs from patient to patient. And as I said, especially at the beginning it was difficult for me to implement all the control, pain control and delirium control well. (A07, item 31)
	22	So I have to say, from a palliative care point of view, I didn't feel overwhelmed. For me, the excessive demands were more on the medical side, i.e. the reasons why a situation is now really palliative. That you can explain them well so that people can go along with you because they then realise it. I found that rather difficult. Otherwise, I found that having a team behind you, for example, the senior ward doctor or the other team members, meant that you could cope very well with it. (K01, item 14)
**Evaluation of teaching in QB 13**<br>*Problems with concrete implementation in everyday life – Communication*	23	But communicating any findings and communicating bad, how should I put it, life-limiting diagnoses or something like that, I think that works so far. I don't know, I'm sure there are people who could do it better, but I think it's okay. (D01, item 16)
	24	So, of course, it's always difficult when delivering such news, but I feel safe doing it. Of course, this also has something to do with how much you know about the course of the disease from a technical point of view. So that you can perhaps estimate a little bit, what's in store for me. Basically, I don't actually feel helpless, it's part of it for me and I don't really have a problem with it and I actually feel safe. (A06, item 11)
	25	Yes, that has already happened to me and it's mainly with relatives who are very interested in it, which I can understand. You are very often asked about these exact time windows, and that is also the situation that was discussed in the course back then. And I think it's particularly good that you were prepared for this, because I wouldn't have thought that many people would want to know so much about it beforehand if you weren't in this situation yourself. But I have the feeling that if you adopt a certain strategy of giving honest but approximate timescales, you are often much better off than saying nothing at all or promising something. And I can remember that, we discussed it more often back then as part of these teachings, so I feel relatively confident about it. (A04, item 18)
	26	I actually find dealing with relatives much more difficult than dealing with patients, because somehow you can't practise that at all. Relatives are often less understanding than the patients themselves. And I found that was less of an issue during my studies. (K07, item 9)
	27	I definitely use the word death. I'm not at all a fan of saying that somehow he falls asleep or doesn't wake up. (...) Of course I sometimes say the word dying instead of death, but I find this word prefinal quite terrible. At least that's my opinion and I definitely say so. (A02, item 19)

	28	So for the conversations themselves, I already have them. I try to do this quite early on, for example when metastases are first diagnosed, so that the disease has changed and we are no longer trying to defeat the disease, but only to keep it in check. So, I use a lot of paraphrases, including paraphrases for death. So, there would be words like "the end" or "in the last stage of life". (D03, item 14)
	29	I try to avoid the word "death" itself. So most of the time I'm talking about death or terminal progression or something similar. (A01, item 13)
	30	And it was precisely these conversations, where you listened or had the conversation yourself, that I think were the most useful, because on the one hand you could see how others do it and deal with it, and on the other hand you could experience yourself in a situation like this without being the one who directly communicates the diagnosis, because it was all more or less an exercise. I've never had to pass on a diagnosis like this myself, but I do think that it helps if you have dialogue training and we've already had that. (K08, item 15)
	31	So, I have gained a lot of impressions, but how I would conduct a conversation now (...). I would say that I can already have a conversation with the patient, but I learnt that more in a professional context, more from copying, so to speak. So I tended to look at my colleagues, my senior physicians, my boss and learnt a bit from them. (D03, item 10)
	32	So, to answer that in two parts: I feel much more confident than when I started my career. So in the last year or year and a half, I've heard a lot of these conversations and had them myself over time, so it's become much better and much safer. (A03, item 12)
	33	So, I still don't find it so easy to talk about. It's also more regulated in our company in such a way that the first breaking bad news is made by the head physician, so to speak. That's not my job then. I then look after the patients as they progress, where they already know that this may lead to death.(D02, item 14)
	34	In general, because we are such a small department, this is more or less not my area of responsibility, because the senior physicians take over. I think I'd get a slap on the wrist too if I did that. (D05, item 15)
**Evaluation of teaching in QB 13**<br>*Problems with concrete implementation in everyday life – dealing with dying*	35	So what we now do quite independently, when we really have patients where we know that they are in the last phase of life, where you can see that they are heading towards prefinal, is that we then add the sedation ourselves (...) even without further help, I would say, or give it to the patient. (...) If I now have a patient who is still clear-headed and it is now a question of him dying in the near future, I have to be honest and say that I am still unsure. (A01, item 15)
	36	Hard to say. I once looked into it myself and read a lot about it. I would now have the feeling that what I know about it, I have rather acquired myself. Although it certainly came up in a lecture, the prefinal, final phase. I do mean that, but not that much. (D02, item 20)
	37	Not really. So about death, yes, issuing a death certificate, but recognising the dying process or dying, we didn't actually deal with that at all during our studies. (A03, item 21)
	38	So yes, I would say that little or nothing was discussed during my studies. And that this is something you learn while working. You also do the first post-mortem together with colleagues. That's also difficult to practise during your studies, it has to be said. But also, in part, how do I recognise that a patient is dying right now? As far as I know or remember, this was not discussed during my studies. (A02, item 31)

Attachment 6 to Dronia MC, Dillen K, Elsner F, Schallenburger M, Neukirchen M, Hagemeier A, Hamacher S, Doll A, Voltz R, Golla H. *Palliative care education and knowledge transfer into practice – a multicenter survey among medical students and resident physicians in Germany using a mixed-methods design*. GMS J Med Educ. 2024;41(3):Doc27. DOI: 10.3205/zma001682

	39	So I think that's something that could be dealt with more. I mean, that's very individual, of course. Some things are the same and others are different. But that's not something that has stuck in my head and I think it's also a lot of gut feeling. Simply, where you get a feeling at some point that you know the person won't survive this or won't live another week or so. But I don't feel like I got any help from a lecture or anything. (K04, item 20)
	40	When I had a stressful shift where patients died and especially when someone died suddenly, where I really cried for half an hour in the changing room after talking to the relatives. So you often find yourself in situations where you are under real psychological strain. (AC 01, item 27).
	41	I know that there is such a psychological service for employees. I haven't used it myself yet. I don't know anyone who uses it either. I wouldn't quite know how to find it either. (...) Now such a direct offer would not know it now. Well, there is one, but I wouldn't know how to make use of it. (D03, item 28)
	42	I think, theoretically, there would be the offer at the university hospital that you can talk to psychologists and psychiatrists here. But I don't think anyone has realised that yet. Neither do I myself and I have often been confronted with stressful situations. (A01, item 27)
	43	So that's a difficult topic. So we're already talking, I have certain cases in mind, we've talked about it a lot in the team. So simply with the colleagues themselves. One case that was particularly close to my heart, I talked about it with friends or called my parents once because I was just far too exhausted after work. (A02, item 41)
	44	Yes, I think that I would most likely use private resources for this. So, of course, there are individual, fateful stories that you tell your friends and family, which is actually a good way of co-operating. So I have to say, I can distance myself from it relatively well, because I still see it as a professional task, where I'm actually more under stress to fulfil the task that also concerns death, so to speak. But if that's not the case, then I would say so. (K01, item 34)
	45	And what I say very clearly and what I also explicitly criticise is that there is actually – not even in our department, where we actually accompany a lot of patients in the dying process and also have critical situations – that there is actually no real offer for a Balint group, for example, or supervision or anything in that area. That there is also no kind of crisis intervention, at least in the medical field – I don't know what it's like in nursing. So someone who is available for this, or at least not that I know of. Someone you could turn to if you were in a difficult situation in your everyday life or something like that. To be honest, I wouldn't know who to turn to. (...) But I think you should actually expect the university hospital and our department to have something like this and to explicitly promote it. (K06, item 35)
	46	I think, or I know from colleagues in other departments, that they tend to sweep it under the carpet. That there is no communication about it, let alone supervision. And that they would rather have a contact point, but they have the feeling that it is not wanted at all in their department or by their superiors. And that is more than a pity and probably leads to a burden that should not exist. So, I definitely see potential for action here. (D06, item 24)
	47	I think palliative medicine is a very practical subject, so I find it a bit difficult in a lecture format. Of course, it's important to learn about the medication and talk about it, because it's also a big part of the doctor's

**Suggestions for improvement in training and education**		job to be able to provide good medical care. (...) I definitely enjoyed my internship with "The Patient as Teacher". But that simply isn't possible for everyone, because there just aren't enough patients. (A02, item 45)
	48	I would like it to be discussed more, but I think the mindset of clinicians is simply that they teach us to heal and not to accompany patients to their death. (...) Even as a young professional, I find it incredible how many friends are shocked when the first death occurs in their home. (DUS 04, item 25).
	49	I hope that one day it will be possible to become a specialist in palliative medicine. And that teaching will definitely be further expanded, as it has been in recent years, so that every medical student can learn about palliative care and ideally also gain practical experience – even if it is only for a few days. And that future doctors are not only taught about dosages and diseases, but that they also get a feel for care structures. (D06, item 28)
	50	So it makes a lot of sense, especially in large hospitals/university clinics, to have a dedicated palliative medicine department with a chair. Perhaps that further training is also offered for doctors. For example, that you say that the drug therapy of pain and shortness of breath should be discussed and that you have a certain guideline. (A04, item 28)
**Visions for the future development of palliative care in Germany**	51	Well, the SAPV in Aachen has very long waiting times. This means that sometimes I can't discharge the patients home if the GPs can't do it, and then they simply suffer with us because we have to wait for several weeks. And then the patients die in hospital or are there for weeks and I think that's a shame when the patients actually wanted something else. (A05, item 38)
	52	And that's what I would like to see for all patients, that everyone who needs it is looked at with a palliative eye to see if there is a need, and if not, it's fine. For example, with chronic heart disease. And perhaps even as early as possible. (D05, item 33)
	53	So perhaps a little more lobbying. That palliative care is perhaps also a little more in focus, which also involves political decisions relating to medicine/healthcare (...) That somehow the lobby would be a bit stronger and that palliative care would perhaps be focussed on even more as a simple service for the chronically ill. And what bothers me again and again is, for example, not that it bothers me, but that as a patient you have to decide whether you want to undergo therapy or whether you want to receive palliative care. And I believe that there are a lot of therapies that would benefit from receiving care at the same time. (D03, item 32)
	54	So in terms of direct patient care, I would like to see the budget for palliative care increased, especially in outpatient care. We often have the problem that we would like to make palliative medical care available to patients earlier and more comprehensively – at least that's how we feel, and I'm just one of many. In my opinion, most patients are also very open-minded, but the fact that there is a lack of hospice places or that the SAPV team has to travel a long way to get to the patient is a problem. I think it would be great if this were to be further expanded in Germany. (K09, item 40)

	55	I think my attitude is a bit consumed because I have the feeling that palliative care is already very well implemented, very accepted and very advanced in our country. I don't think that's the case everywhere. On the one hand, not at all clinics, and on the other, not in all specialist disciplines. So the collaboration between oncology and palliative care is already much more advanced than in other specialist disciplines. And I would hope, I would imagine, that this would be applied to other clinical pictures, other specialist disciplines, that there would be a similar realisation that this is an important part of treatment and that there would be room for it and that it would be accepted. There is also the well-known example that cardiac insufficiency or similar conditions have a very poor prognosis, and there would probably be a similar need for treatment as for oncological diseases, but this has not yet been covered in this way. (K05, item 35)
	56	And perhaps the general population would also be taught a little more about palliative care, because I used to think that before I went to university: "Okay, a hospice is a place where people go to die, it has a really oppressive atmosphere, it's really bad." That's how I always imagined a hospice. And now I think very differently about it. I then think of a place that is not about time in the sense of days, but about quality that takes place within this time, and that can be something very enriching. (K09, item 41)
